# The Advancing Understanding of Transportation Options (AUTO) study: design and methods of a multi-center study of decision aid for older drivers

**DOI:** 10.1186/s40621-021-00310-4

**Published:** 2021-05-03

**Authors:** Marian E. Betz, Faris Omeragic, Lauren Meador, Carolyn G. DiGuiseppi, Nicole R. Fowler, S. Duke Han, Linda Hill, Rachel L. Johnson, Christopher E. Knoepke, Daniel D. Matlock, Ryan Moran, on behalf of the AUTO Research Team, Miles Breese, Abigail Evans, Alexa Hansen, Eleanor Batista-Malat, Natalie Moursund, Alicia Okimura, Sarah Andrade, Eugenia Orehova, Shelley Suarez, Ruby Vianzon, Anna Williams

**Affiliations:** 1 Department of Emergency Medicine, School of Medicine, University of Colorado Anschutz Medical Campus, Aurora, CO USA; 2 VA Eastern Colorado Geriatric Research Education and Clinical Center, Aurora, CO USA; 3 Department of Epidemiology, Colorado School of Public Health, University of Colorado Anschutz Medical Campus, Aurora, CO USA; 4 Center for Aging Research, Indiana University School of Medicine, Regenstrief Institute, Indianapolis, IN USA; 5 Department of Family Medicine, University of Southern California, Los Angeles, CA USA; 6 School of Public Health, University of California San Diego, San Diego, CA USA; 7 Department of Biostatistics and Informatics, Colorado School of Public Health, University of Colorado Anschutz Medical Campus, Aurora, CO USA; 8 Adult & Child Consortium for Outcomes Research & Delivery Science, School of Medicine, University of Colorado Anschutz Medical Campus, Aurora, CO USA; 9 Division of Cardiology, School of Medicine, University of Colorado Anschutz Medical Campus, Aurora, CO USA; 10 Division of Geriatric Medicine, Department of Medicine, University of Colorado School of Medicine, Aurora, CO USA

**Keywords:** Older driver, Driving, Decision-making, Motor vehicle, Randomized trial, Geriatric, Decision aid

## Abstract

**Background:**

Decision-making about when to stop driving for older adults involves assessment of driving risk, availability of support or resources, and strong emotions about loss of independence. Although the risk of being involved in a fatal crash increases with age, driving cessation can negatively impact an older adult’s health and well-being. Decision aids can enhance the decision-making process by increasing knowledge of the risks and benefits of driving cessation and improve decision quality. The impact of decision aids regarding driving cessation for older adults is unknown.

**Methods:**

The Advancing Understanding of Transportation Options (AUTO) study is a multi-site, two-armed randomized controlled trial that will test the impact of a decision aid on older adults’ decisions about changes in driving behaviors and cessation. AUTO will enroll 300 drivers age ≥ 70 years with a study partner (identified by each driver); the dyads will be randomized into two groups (*n* = 150/group). The decision aid group will view the web-based decision aid created by Healthwise at baseline and the control group will review information about driving that does not include evidence-based elements on risks and benefits and values clarification about driving decisions. The AUTO trial will compare the effect of the decision aid, versus control, on a) immediate decision quality (measured by the Decisional Conflict Scale; primary outcome); b) longitudinal psychosocial outcomes at 12 and 24 months (secondary outcomes); and c) longitudinal driving behaviors (including reduction or cessation) at 12 and 24 months (secondary outcomes). Planned stratified analyses will examine the effects in subgroups defined by cognitive function, decisional capacity, and readiness to stop driving.

**Discussion:**

The AUTO study is the first large-scale randomized trial of a driving decision aid for older adults. Results from this study will directly inform clinical practice about how best to support older adults in decision-making about driving.

**Trial registration:**

ClinicalTrials.gov: NCT04141891. Registered on October 28, 2019. Located at https://clinicaltrials.gov/ct2/show/NCT04141891

**Supplementary Information:**

The online version contains supplementary material available at 10.1186/s40621-021-00310-4.

## Background

There are 44 million licensed drivers aged ≥65 years in the United States (CDC 2020) and driving remains the primary mode of transportation for older Americans (Choi et al. 2012). Driving is linked to individual well-being and driving cessation can negatively impact older adults independence and mental health (Chihuri et al. 2016; Edwards et al. 2009; Foley et al. 2002; Harmon et al. 2018). Identifying ways to support older drivers to stay on the road safely and maintain independence and community involvement is a national priority (Classen et al. 2007; NHTSA 2010; NHTSA 1999). Safety is an important consideration since older drivers are at increased risk of crashes (Classen et al. 2007; Pomidor 2016). Fatal crash rates among older drivers increase after age 75 (Cicchino 2015); generally older drivers pose a greater risk to themselves than to the community around them (Braver and Trempel 2004; Tefft 2008). Estimation of an individual driver’s risk remains difficult, as on-road testing is costly and not always available (Betz et al. 2014) and office-based assessments are impractical (Betz et al. 2015) and not routine (Betz et al. 2016a).

Decisions about changing driving habits or cessation are difficult and emotionally-laden, owing to intersecting implications of safety, independence, and personal well-being. Additionally, these decisions generally involve multiple people, including the older driver and family members or trusted friends. In some instances, healthcare providers are involved at the request of family or due to concerns about safety. Older drivers fear losing their independence, being abandoned, or becoming a burden on others, while simultaneously fearing causing harm to others on the road (Betz et al. 2016b). Decisions about driving are complicated by older adults’ functional ability, cognitive ability, decisional capacity, personality, and other factors such as financial resources or access to alternative transportation options. Of particular importance is understanding the impact of cognitive impairment and driving decisions. While physical function and certain medications can affect driving quality, Alzheimer’s disease and other forms of progressive cognitive impairment may have the strongest link to both driving risk and the need for eventual driving cessation (Carr and Ott 2010; Pomidor 2016). Given the estimate of almost 16 million older adults in the US with dementia by 2050 (Alzheimer’s Association 2020), the decision-making needs around driving for this group is significant. The role of family members, trusted friends, and healthcare providers in supporting a driver through driving retirement gains additional importance in the context of cognitive impairment or concerns about decisional capacity.

Guides and self-assessment tools exist to help older drivers and their families think about driving (AAA 2005; NIA 2014; Pomidor 2016; The Hartford 2010), but they do not include evidence-based elements that assist with values clarification and assessing the risks and benefits of driving cessation. Healthwise, a nonprofit organization that provides decision support tools and other services to enhance patient-centered decision making, released a decision aid in 2015 for US drivers with and without cognitive impairment (Healthwise 2016). The tool was developed according to international decision aid standards (Coulter et al. 2013; OHRI 2015), is available online, and is accessible to clinicians in healthcare systems who use Healthwise tools, about 25% of clinicians in the United States. However, the Healthwise decision aid has never been tested in a randomized controlled trial to evaluate its effectiveness on decision quality or person-centered outcomes.

## Methods

### AUTO study design

The Advancing Understanding of Transportation Options (AUTO) study is a randomized, controlled trial being conducted with older primary care patients in three US states that seeks to assess the effects of the Healthwise decision aid among older adults and a study partner (SP). AUTO will test the decision aid in improving decision making and quality and determine its effects on specific subpopulations of older drivers defined by cognitive function, decisional capacity, and attitudes about mobility transition. The hypothesis is that the decision aid will improve the quality of decision making about driving behaviors, which will mitigate the negative psychosocial impacts of driving reduction or cessation. The AUTO trial will enroll 300 patient-study partner dyads from primary care clinics in California, Colorado, and Indiana, USA. These dyads will be randomized into two groups (*n* = 150), stratified by site. Both members of the dyad review the same site (decision aid or control). Dyads randomized to the Healthwise driving decision aid will view the decision aid, separately, immediately following baseline assessment (Healthwise 2016). Dyads randomized to the control group view the National Institute on Aging (NIA) “Older Drivers” website (NIA 2014) immediately following the baseline assessment. Dyads in both groups are instructed to view the decision aid or control site at their own pace and navigate through the site components at their own discretion. The AUTO trial will measure the primary and secondary outcomes at baseline, 6, 12, 18 and 24 months. Consent, enrollment, data collection, and viewing the decision aid or control material is done by telephone and access to the internet; prior to the COVID-19 pandemic, it was also done face-to-face (see below).

This study protocol has followed the Standard Protocol Items: Recommendations for Interventional Trials (SPIRIT) Guidelines (Chan et al. 2013). The trial will be conducted and reported according to the reporting of pragmatic trials: an extension of the Consolidated Standards of Reporting Trials (CONSORT) Statement. The study has been approved by the institutional review boards of University of California San Diego, University of Colorado, and Indiana University. The AUTO trial is registered with clinicaltrials.gov (Clinical Trials.gov Identifier NCT04141891).

Older adult-family member dyads are recruited from primary care clinics affiliated with each study, with an enrollment goal of 100 drivers and ≥ 67 study partners (i.e., ≥67 study dyads) per site.

### Eligibility

The target population is dyads formed by: (1) an adult aged 70 or older (*n* = 300) and (2) a family member, friend, or legal healthcare power of attorney whom the patient identifies as someone who might be involved in decision-making about driving or in providing support for the transition to non-driving (*n* = up to 300; Table 1). Eligibility for patients is established through screening of the patient’s electronic health record (EHR) and by assessments conducted by the research assistants face-to-face or via the telephone. The study seeks to enroll drivers more likely to be primed to consider driving retirement, so an eligibility criterion is that that they have ≥1 diagnosis of a progressive medical condition associated with reduced driving ability and increased risk of cessation, as defined by our study team (Appendix 1). These conditions were abstracted from participant’s electronic medical record list of diagnoses and verbally confirmed during eligibility screening.

**Table 1:** Eligibility criteria for AUTO study

Inclusion	Exclusion
Fluent in English Have a telephone number for follow up interviews Age: ≥70 years (drivers); ≥18 years (study partners) Drivers only: Valid driver’s license from study site’s state Drive at least once a week Since the last license renewal, no major changes to health, vision, or hearing that seriously impair driving (self-report) Do not feel the DMV would have serious concerns about driving (self-report) Have ≥1 medical condition linked in driving cessation (EMR, confirmed by self-report; see Appendix)	5-min Montreal Cognitive Assessment (MoCA) score < 21 In legal custody or institutionalized Drivers only: Currently enrolled in LongROAD longitudinal study (also at UCH and UCSD)

### Recruitment

Site study coordinators obtain lists of potentially eligible older driver participants at each site and then mail recruitment letters with a site-specific recruitment flyer. If the participant has not reached out to the site, a phone call is made two weeks after the letter is sent to inquire about interested and eligible. If interested and eligible, the older driver is asked for the name and contact information of a family member and additionally requests permission to call the family member to assess if they are also eligible and willing to enroll. Eligibility screening for the study partner is conducted prior to consenting the older driver. If the older driver does not identify a study partner or the study partner is ineligible or not interested, the older driver is placed on a waitlist to be contacted for enrollment once dyadic recruitment goals are met.

The AUTO trial will reduce loss to follow-up for longitudinal assessments by engaging the older drivers every 6 months throughout the study (6, 12, 18, and 24 months) and SPs every 12 months (12 and 24 months), including keeping the assignment of research staff and participants consistent at each outcome assessment and sending reminder letters.

### Randomization and blinding

Enrolled older drivers are randomized in blocks to reduce bias and aim for balance among arms (Efird 2011), with randomly varied block sizes of 4 and 6, in a 1:1 ratio of intervention to control group. The allocation is concealed using a centralized, computer-generated list that study coordinators access once the RA completes the administration of pre-randomization measures. The driver and SP are randomized to the same arm but complete study measures and interventions on their own. Participants are instructed to view the intervention or control site at their own pace and by navigating through the site components at their own discretion. After completing the decision aid or website review, all participants in both arms answer questions about knowledge, values, and driving retirement plans.

Participants are blinded to their allocation, though they know that the study is about driving. RAs cannot be blinded to the baseline assignment of SPs since SPs are given the same assignment as the driver. Whenever possible, a different RA conducts follow-up interviews to be blinded to study arm.

### Description of intervention

The Healthwise decision aid is for older adults considering the decision “Is it time to stop driving?” (Healthwise 2016). The online decision aid has six sections: “Get the Facts,” “Compare Options,” “Your Feelings,” “Your Decision,” “Quiz Yourself,” and “Your Summary.” The decision options (“Stop driving” or “Keep driving”) are presented with their benefits and risks or side effects, alongside personal stories from other adults facing the decision. The “Your Feelings” page allows users to rate on 7-point Likert scales their: concern about getting into an accident; comfort while driving; fear of harming others; concern from others; and ability and willingness to use other sources of transportation. The “Your Decision” prompts them to rate (on a 7-point Likert scale) their current plan, from “leaning toward stopping driving” to “leaning toward keeping driving.” The online DDA has a simple greyscale interface without images or videos.

### Description of control

The NIA “Older Drivers” website (NIA 2014) was chosen as a control because it best represents easily-accessible and freely-available information about driving risk and driving cessation that any older adult with internet access could view. However, the NIA website does not guide the individual through the decision-making process. It includes a personal story from an older adult, information about various medical conditions that can affect driving, and ideas of alternative transportation. It does not include videos or images.

### Theoretical framework

**Fig. 1:**
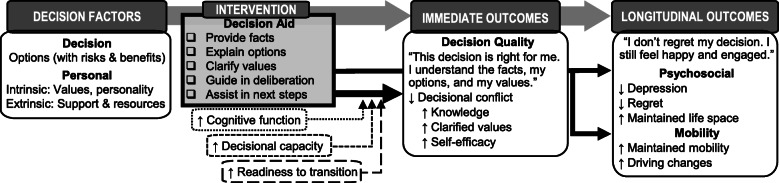
Theoretical framework of decision aid’s immediate and long-term impact. Adapted from Ottawa Decision Support Framework (O’Connor 2006)

Key measures in this trial are tied directly to the Ottawa Decision Support Framework (O’Connor 2006), which posits that decisional needs (e.g., knowledge, conflict/uncertainty, and values) affect decision quality (Fig. 1). High quality decisions, which are those both informed and reflective of the individual’s values, can spur action and subsequent health outcomes and patient’s feelings about the decision, such that the highest quality decision will have the best outcome for the patient. Decision aids, including the Healthwise one being evaluated in AUTO, generally have four key sections: (a) identify the decision to be made, (b) describe risks and benefits of various options, (c) assist the individual in clarifying personal values, and (d) activate the individual for decision-making (Bhandari et al. 2008; Matlock and Spatz 2014).

### Primary and secondary outcome measures

The primary immediate outcome is decision conflict. Secondary, longitudinal outcomes are psychosocial- and mobility-related (Fig. 1; Table 2). Primary and secondary outcome measures will be assessed at baseline, 6, 12, 18, and 24 months by blinded research assistants, with planned analyses stratified by subgroups. No data is collected until informed consent are obtained. Full study measures are shown in Table 2.

**Table 2:** Overall study flow for older drivers (D) and study partners (SP), following SPIRIT template of recommended content for the schedule of enrollment, interventions, and assessments (Chan et al. 2013)

	STUDY PERIOD							
	Enrollment	Allocation		Post-allocation				Close-out
**TIMEPOINT (months)**	***-t***	**t**_**0**_	**t**_**0**_	**t**_**0**_	***t***_***6 mo***_	***t***_***12 mo***_	***t***_***18 mo***_	***t***_***24 mo***_
**ENROLLMENT**								
Eligibility screen	X^D,SP^							
Informed consent		X^D,SP^						
Randomization			X^D,SP^					
**INTERVENTIONS**								
Driving decision aid			X^D,SP^					
Control website			X^D,SP^					
**ASSESSMENTS**								
**Immediate outcomes**								
Decision Conflict Scale		X^D,SP^		X^D,SP^	X^D^	X^D,SP^	X^D^	X^D,SP^
Knowledge questionnaire		X^D^		X^D,SP^	X^D^	X^D,SP^	X^D^	X^D,SP^
Values concordance (DCS Values Clarity subscale)		X^D,SP^		X^D,SP^	X^D^	X^D,SP^	X^D^	X^D,SP^
Decision Self-Efficacy scale		X^D^		X^D,SP^	X^D^	X^D,SP^	X^D^	X^D,SP^
**Longitudinal outcomes**								
PROMIS Depression		X^D^			X^D^	X^D^	X^D^	X^D^
Ottawa Decision Regret Scale					X^D^	X^D,SP^	X^D^	X^D,SP^
Life-Space Assessment		X^D^			X^D^	X^D^	X^D^	X^D^
Driving behaviors		X^D^			X^D^	X^D,SP*^	X^D^	X^D,SP*^
**Subgroups**								
Cognitive screening (5-min MoCA)	X^D,SP^				X^D^	X^D,SP^	X^D^	X^D,SP^
Cognitive function (BTACT, OTMT)		X^D^						
Beck Cognitive Insight Scale		X^D^						X^D^
Decisional capacity (SPACED)		X^D^						X^D^
Attitudes about driving (ARMT)		X^D^			X^D^	X^D^	X^D^	X^D^
Driving behaviors		X^D,SP^			X^D^	X^D,SP*^	X^D^	X^D,SP*^
**Other covariates**								
Demographics		X^D,SP^			X^D^	X^D,SP^	X^D^	X^D,SP^
Physical health		X^D^			X^D^	X^D^	X^D^	X^D^
Mental health		X^D^			X^D^	X^D^	X^D^	X^D^
Personality (TIPI)		X^D^						
Driving education		X^D,SP^			X^D^	X^D,SP^	X^D^	X^D,SP^
Family questionnaire		X^SP^				X^SP^		X^SP^
Major life events					X^D^	X^D,SP*^	X^D^	X^D,SP*^
COVID-19 related health				X^D,SP^	X^D^	X^D,SP^	X^D^	X^D,SP^

*Questions about older driver, as answered by study partner

#### Primary outcome

The primary outcome is decision quality as estimated by the Decisional Conflict Scale (DCS; Appendix 2)(O’Connor 1993; Sepucha et al. 2013). Decision quality is a fundamental element of the Ottawa Decision Support Framework (O’Connor 2006) as a precursor to behavior change, with a high-quality decision defined as an informed patient making a decision consistent with their values (Sepucha et al. 2013). The DCS measures internal conflict or ambivalence about the decision, with higher internal conflict (or ambivalence) indicating the decision is less in-line with personal values. The DCS is a 16-item scale with three subscales, including Values Concordance. The DCS has strong reliability and test-retest correlation (Cronbach’s alpha coefficients > 0.78) and has previously shown to discriminate between known groups who make or delay decisions (effect size 0.4–0.8). Scores < 25 (out of 100 total) have previously been associated with implementing decisions (O’Connor 1993). DCS scores range from 0 (extremely clear) to 100 (extremely unclear about personal values).

Secondary immediate outcomes related to decision quality are knowledge about driving decisions and decision self-efficacy. The Knowledge questionnaire, created for this study, assesses concepts about driving presented in the decision aid and control group websites. The Decision Self-Efficacy Scale will be used to measure participants’ self-confidence or belief in their ability to make decisions about driving (O’Connor 1995), as decision aids can increase self-efficacy. Scores range from 0 (extremely low) to 100 (extremely high self-efficacy).

#### Secondary outcomes

Depressive symptoms are measured using the PROMIS 4-item scale, with higher scores indicating higher depression. All PROMIS scores are analyzed as standardized T-scores (mean = 50, SD = 10) (HealthMeasures 2020).

The Ottawa Decision Regret Scale is a validated measure that correlates with decision satisfaction and conflict; it is scored from 0 to 100, with higher scores representing higher regret (O’Connor 1996).

The Life-Space Assessment instrument (UAB Study of Aging) is a validated tool assessing recent mobility and function (Baker et al. 2003). Composite scores range from 0 (bedbound) to 120 (travel out of town every day without assistance) (Stalvey et al. 1999); scores of ≤60 are correlated with lower levels of social participation and higher mortality (Phillips et al. 2015).

Driving-related measures drawn from prior studies and developed de novo include self-reported driving frequency (days per week), avoidance in certain situations (e.g., night), driving cessation (none, partial, complete), and crashes (≥1 versus none).

#### Cognitive measures

Overall cognitive status is assessed through the initial screening 5-min MoCA (Wong et al. 2015), the Brief Test of Adult Cognition by Telephone (BTACT), and the Oral Trail Making Test (OTMT) (Lachman et al. 2014; Mrazik et al. 2010). A study neuropsychologist uses these cognitive tests to categorize participants’ cognitive function as no impairment, mild cognitive impairment, or dementia based on the BTACT Composite and OTMT-B (z-score < − 1.5 is impaired).

The Beck Cognitive Insight Scale combines sub-scales (self-reflectiveness and self-certainty) into a composite index, with lower scores indicating lower insight (Beck et al. 2004).

The Short Portable Assessment of Capacity for Everyday Decision-Making (SPACED) measures decision-making capacity (Lai et al. 2008). There are four criteria, each scored with a 0 for inadequate, 1 for marginal, and 2 for adequate, producing a total possible range of 0 to 8.

The Attitudinal Readiness for Mobility Transitions (ARMT) measures affective and emotional aspects of present or future mobility changes associated with cessation (Meuser et al. 2013). It has four subscales: anticipatory anxiety, perceived burden, avoidance, and adverse situation (i.e., the view that mobility loss is harmful to quality of life). Each of the 24 items is rated on a 1–5 Likert scale, and higher total average scores indicate lower readiness to transition (Meuser et al. 2013).

#### Other measures

Baseline and follow-up Questionnaires assess demographic characteristics and Activities of Daily Living. PROMIS measures include Global Health V1.2, Emotional Support (4-item), and Social Isolation (4-item) (HealthMeasures 2020). Additional measures are the 4-item Perceived Stress Scale (Cohen et al. 1983) and the 10-Item Personality Inventory (Gosling et al. 2003).

Follow-up questionnaires assess exposure to print or non-study online materials about driving safety or cessation, as these may affect decisions about driving cessation, as well as major life events since last contact.

Questions drawn from the The CoRonavIruS Health Impact Survey (CRISIS V0.1) Adult Self-Report Baseline Current Form (Bromet et al. 2021) were added to the questionnaires in spring 2020 to assess how the COVID-19 pandemic was affecting participants’ well-being and mobility. Study staff also began tracking relevant local orders that might limit driving (e.g., stay-at-home orders).

At baseline and follow-up, SPs complete additional questions about their relationship with the driver.

### Data monitoring

The data safety monitoring plan (DSMP) for this trial includes monitoring by the PI and a Data Safety and Monitoring Board (DSMB). The DSMB Charter contains a detailed list of the DSMB responsibilities. The DSMB will act in an advisory capacity to the IRB and NIA Program Official in order to monitor participant safety, evaluate the progress of the study, and review procedures for data management and analysis, maintaining the confidentiality of data, and the quality of data collection.

Potential adverse events that will be monitored in the AUTO study include: death (any reason), inpatient hospitalization (any reason), emergency department visit (any reason), or motor vehicle crash. Each adverse event is graded by severity and relationship to intervention.

Given concerns over the safety of older drivers and the community around them, the AUTO team developed specific procedures related to potentially-impaired drivers. For older drivers with an initial 5-min MoCA score of 21–25 (Additional file 1), study staff review questionnaire elements related to the American Academy of Neurology guidelines (Iverson et al. 2010). If the driver has ≥3 risk factors (e.g., reported crashes), the site PI contacts the driver to recommend they have a professional driving evaluation and talk with their primary care provider. The driver is allowed to continue participation in the study whether or not they complete a driving evaluation. Additionally, AUTO staff can note concerns from participant interactions, such as confusion with questions or suspected memory problems in participants. These concerns similarly prompt an ad hoc review and potential contact of the older driver by the site PI with a recommendation to talk to their physician or trusted family or friends (Additional file 2). All these procedures are explained during the informed consent process. At the time of consent, older drivers are asked if they want to provide optional consent for the research team to contact their primary care provider or a designated friend or family member about their driving safety, should the team have concerns about safety.

### Data collection

Before March 2020, RAs met with older drivers to complete the baseline visits in person at on-campus sites or participants’ homes. The baseline session includes: written informed consent including HIPAA authorization and permission to access driver licensing records (crash records for the past 12 months before enrollment and up to every 12 months after enrollment); administered questionnaires before and after randomization; brief cognitive tests; and viewing intervention or control information on a tablet. Study measures and point of administration are shown in Fig. 1. Procedures for SPs are similar, although SPs could complete their baseline session either in-person or over the phone. Response cards were used to help facilitate the various questionnaires.

The COVID-19 pandemic and campus closures prompted a change in the study protocol such that all older driver and SP enrollments are done by phone. Baseline sessions completed by telephone (unless the participant requested Zoom videoconference) use a postcard consent with a waiver of written documentation. Response cards and a link to the website (decision aid or control) are emailed to participants, with instructions to view them on the participant’s desktop, laptop, tablet, or smart phone.

Participants are contacted for telephone follow-up at pre-specified intervals: 6, 12, 18, and 24 months after initial visit for drivers; 12 and 24 months after initial visit for SPs. Participants are contacted via phone call or email (depending on participants’ preference) to schedule the follow-up interviews. Trained research staff follow all institutional review board (IRB) policies regarding contacting participants, with at least 3 but no more than 10 contact attempts for non-responders at each follow-up interval. Research staff attempt to call participants at different times of day, leaving brief messages on varying attempts, and contact participants via email and mail as needed.

RAs at each site enter all data into a secure research database (REDCap, Research Electronic Data Capture) (Harris et al. 2009). Data access privileges specify only those privileges required by each individual in their specific organizational role, so RAs can only see their site’s data, and limited study team members (PI, biostatistician, and analyst) can see data from all sites. All study documents are stored in locked cabinets or password-protected files on secure university servers. Recruitment, enrollment, and follow-up reports are sent weekly to research staff, coordinators, and co-investigators to allow monitoring of study progress.

### Timeline

The recruitment of patients began in December 2019, with anticipated enrollment completion by June 2021 and all data collected from recurring outcome assessments expected to be collected by June 2023.

### Analysis plan

Planned analyses will be performed according to the principle of intention-to-treat, including all randomized drivers. Unless otherwise specified, hypothesis tests will be two-sided with alpha = .05, with 95% confidence intervals or *p* values reported. Descriptive statistics will be computed for baseline patient characteristics, initially testing for differences between control and intervention groups. Site effects will be assessed by comparing demographic variables across the sites, using one-way ANOVA tests for continuous measures and chi-square tests of proportions for categorical measures. If there are significant differences among sites, then (assuming enough events) analysis will use separate models for each aim described below, with a fixed effect for site and with treatment arm as the main predictor. For longitudinal analyses, continuous and logistic outcomes will be modeled using generalized estimating equations with unstructured correlation structure to account for repeated observations on each participant. Analyses of the decision aid’s longitudinal effects may be vulnerable to bias, as control arm participants may be exposed to intervention arm messages through exposure to available materials or courses related to driving safety or cessation (e.g., websites, physician counseling). Analyses may adjust measurements of contamination as applicable. In all analyses, validation of distributional and parameterization assumptions will be checked and data transformations (e.g. log-transformations) or alternative methods will be implemented as appropriate.

Planned analyses will first test the effect of a web-based decision aid as compared to control (web-based information only) on: (a) immediate decision quality, hypothesizing that more decision aid participants will make high-quality decisions; (b) longitudinal psychosocial outcomes at 12 and 24 months, hypothesizing that decision aid participants will have reduced prevalence of depressive symptoms and of decision regret but maintained life space; and (c) longitudinal driving behaviors at 12 and 24 months, hypothesizing that the decision aid—although not intended to direct participants to continue or stop driving—will lead to changes. Next, stratified analyses will determine the decision aid’s effects in specific subpopulations, including: (a) older drivers with versus without cognitive impairment, hypothesizing that the decision aid will improve decision quality more in cognitively intact drivers; (b) older drivers with maintained versus impaired decisional capacity, hypothesizing that the decision aid will improve decision quality more in drivers with maintained decisional capacity; and (c) older drivers who are attitudinally more versus less ready for a mobility transition, hypothesizing that the decision aid will improve decision quality more in drivers who are ready for transition.

Study partner data will be analyzed for the primary outcome (DCS) with regression models that allow assessment of marginal effects of the intervention on older drivers and partners separately while jointly accounting for the correlation between drivers and partners, as well as their concordance/discordance in decision quality. We will also examine the degree of concordance (or discordance) within driver-partner dyads on various measures, including current versus desired level of partner involvement in driving retirement process (part of the driving questionnaire).

Study scales and variables were chosen carefully to keep questionnaires as short as possible and minimize overlap. To avoid inflation of our type I error rate, we selected DCS as our single primary endpoint that will be tested to assess the overall efficacy of the intervention. Other secondary outcomes (as listed above) will be considered subsidiary and exploratory rather than confirmatory. (Li et al. 2017b).

### Sample size and power analysis

The target sample size (*n* = 300; 150 intervention and 150 control) was chosen to allow detection of a 20–40% difference between the decision aid and control arms (depending on underlying proportions for each of the treatment arms) for the primary outcome (DCS score < 25) at a power of 90% and a 0.05 significance level, while allowing for 10% loss to follow-up. The overall sample size also allows for stratified analyses. Estimates for the effect of the driving decision aid on behavior among older drivers do not exist, so the assumption of a 20–40% difference is conservative (prior work with other DAs has found an effect size of 40–80% on DCS between groups) (O’Connor 1993).

## Discussion

The AUTO study is the first large-scale trial in the United States for a decision aid for driving retirement among older adults. Its randomized design allows examination of the effect of a decision aid on both immediate and long-term outcomes, its multi-site recruitment will improve generalizability, and its longitudinal follow-up will complement existing observational studies of driving behaviors among older adults, like the LongROAD study and Candrive/Ozcandrive (Li et al. 2017a; Marshall et al. 2013).

The COVID-19 pandemic arose shortly after study enrollment began, prompting a shift to completely remote enrollment as of March 2020. This posed certain logistic challenges related to the length of the interview (which was more tiring for participants by telephone than in person) and technical difficulties in setting up the response cards and intervention on a home device during the baseline phone call. An additional challenge was that during the period of remote enrollment, a greater proportion of potential participants did not answer phone calls, perhaps because the call-back number is not identified as a university-affiliated number. However, the shift to remote study activities brought positive effects as well. This included a more rapid rate of enrollment and high rates of participation among those eligible, perhaps related to the desire of older adults to be engaged during times of social distancing or quarantine (Fuller and Huseth-Zosel, 2021).

The nature of the study raised a unique challenge related to the tension between study integrity and participant safety. The study seeks to examine the factors affecting an older adult’s decision about driving, with a desire to avoid providing information, guidance, or recommendations to participants (other than the decision aid itself for the group). Yet responses to study measures or interactions with staff might uncover cognitive or physical issues that have the potential to impair driving ability, thereby posing a risk to the participant and those around them. In addition, the study specifically seeks to enroll older adults with at least one medical condition (including mild cognitive impairment) that might contribute to driving retirement so as to allow an adequate sample of older adults who stop driving during the study. The AUTO team, in consultation with its DSMB, developed study protocols and safety checks to protect participant safety and well-being without overly interfering in decision-making about driving retirement.

Additional study limitations include challenges, and potential biases, in enrolling study dyads. Specifically, some older adults may not have or want to suggest a family member or friend to participate as a study partner, and the study design may not allow a large enough sample of these older drivers (without partners) for detailed subgroup analyses. We chose to allow some older adults to enroll without a partner out of recognition that their experiences and outcomes may differ from those with a partner, and the study will provide at least preliminary analyses. We chose to have participants and partners complete the decision aid separately, so we could measure their responses separately (and potential concordance); in real life, such tools may be used together. The COVID-19 pandemic itself affected older drivers’ behaviors (Morrow-Howell et al. 2020; Rantanen et al. 2020), with reduced frequency and distances driven and anxieties about the safety of public transportation, which might impact the study’s analyses and outcomes. Added study questions about COVID-19 and its effects on mobility and on mental and physical health will allow examination of some of these issues. In addition, internet access was needed to view the informational website and response option cards in real time, which may have affected participant diversity.

## Conclusion

The AUTO study has the potential to help fill knowledge gaps concerning decision making about driving retirement and to advance the science and practice of safe mobility through a life-course perspective. The innovative application of the decision aid model to older driver decision making offers the possibility of facilitating decisions about driving retirement in a person-centered, acceptable, feasible way, and consequently it has the potential to reduce the negative psychosocial outcomes associated with driving retirement. Understanding whether and with whom to use a driving decision aid has the potential to significantly improve the independence, health, and well-being of millions of older adults.

### Supplementary Information

**Additional file 1: Risk Review Form (based on AAN Guidelines** (Iverson et al. 2010).

**Additional file 2: Cognitive Concern Form**.

## Data Availability

Requests for data from other researchers and the public will be considered, and data will be made available in accordance with local institution policies, IRB recommendations, local/state/federal laws and regulations, and considerations for publication. Any applicable data sharing will follow HIPAA rules.

## Appendix 1

**Table 3:** Medical conditions, as identified in electronic medical record, with ≥1 required for older driver eligibility

Medical Condition	Examples
Diseases/conditions affecting vision	Diabetic retinopathy
	Macular degeneration
	Glaucoma
	Retinitis pigmentosa
	Field cuts
	Low visual acuity even after correction
Cardiovascular disease, especially when associated with presyncope, syncope, or cognitive deficits	Unstable coronary syndrome
	Implantable defibrillator
	Congestive heart failure
	Hypertrophic obstructive cardiomyopathy
	Orthostatic hypotension
	Syncope or presyncope
Neurologic disease	Narcolepsy
	Dementia
	Multiple sclerosis
	Parkinson disease
	Brain injury
	Spinal cord injury
	Stroke
	Vertigo or dizziness
	Seizure
Psychiatric disease	Alcohol or other substance abuse
Metabolic disease	IDDM
Musculoskeletal disabilities	Arthritis and foot abnormalities
Respiratory disease	Chronic obstructive pulmonary disease
	Obstructive sleep apnea
Chronic renal failure	End Stage Renal Disease
	Hemodialysis
Insomnia	Sleep apnea
	Insomnia
	Restless leg syndrome

## Appendix 2

### **Decision Conflict Scale (DCS)** (O’Connor 1993)

When you think about driving, which way are you leaning? Please use a 1 to 7 scale where 1 is Leaning Toward Stop Driving, 4 is Undecided, and 7 is Leaning Toward Continue Driving.

**Table:**

Stop Driving						Continue Driving
1	2	3	4	5	6	7
Leaning Toward			Undecided			Leaning Toward

In thinking about your preference for whether to stop or continue driving, please indicate how strongly you disagree or agree with the following statements using a 1 to 5 scale where 1 is Strongly Disagree and 5 is Strongly Agree.

**Table:**

***Statement***	Strongly Disagree	Disagree	Neither Agree Nor Disagree	Agree	Strongly Agree	Don’t Know
I know which options are available when it comes to stopping or continuing driving.	1	2	3	4	5	88
I know the benefits of stopping or continuing driving.	1	2	3	4	5	88
I know the risks or drawbacks of stopping or continuing driving.	1	2	3	4	5	88
I am clear about which benefits matter most to me.^VC^	1	2	3	4	5	88
I am clear about which risks or drawbacks matter most to me.^VC^	1	2	3	4	5	88
I am clear about which is more important to me (the benefits or the risks and drawbacks). ^VC^	1	2	3	4	5	88
I have enough support from others to make a choice when it comes to stopping or continuing.	1	2	3	4	5	88
I am choosing to either stop or continue driving without pressure from others.	1	2	3	4	5	88
I have enough advice to make a choice to stop or continue driving.	1	2	3	4	5	88
I am clear about the best choice for me.	1	2	3	4	5	88
I feel sure about what to choose from.	1	2	3	4	5	88
The decision to stop or continue driving is easy for me to make.	1	2	3	4	5	88
I feel I have made an informed choice.	1	2	3	4	5	88
My decision shows what is important to me.	1	2	3	4	5	88
I expect to stick with my decision to stop or continue driving.	1	2	3	4	5	88
I am satisfied with my decision to stop or continue driving.	1	2	3	4	5	88

(^VC^=Values Clarity Subscale)

## Abbreviations

AUTO: Advancing Understanding of Transportation Options
DCS: Decisional Conflict Scale
DSMB: Data Safety and Monitoring Board
IRB: Institutional Review Board
MoCA: Montreal Cognitive Assessment
NIA: National Institute on Aging
RA: Research Assistant
SP: Study Partner
