# Supplementary material for: The Advancing Understanding of Transportation Options (AUTO) study: design and methods of a multi-center study of decision aid for older drivers
Source: Inj Epidemiol. 2021 May 3;8:23. doi: 10.1186/s40621-021-00310-4 (PMC8088834; doi:10.1186/s40621-021-00310-4)
Supplement: Supplementary file 1 — Additional file 1: Risk Review Form (based on AAN Guidelines (Iverson et al. 2010). [file 40621_2021_310_MOESM1_ESM.docx]

**Appendix 3. Risk Review Form (based on AAN Guidelines** (Iverson et al. 2010)

***INSTRUCTIONS:*** This form should only be filled out for Drivers who have a recently completed MoCA score less than or equal to 25.

Were the AAN risk factor REDCap reports reviewed for this time-point?

🞏 Yes

🞏 No

Date of review _______________

Who performed the review? _______________

Were 3 or more AAN risk factors identified for this participant?

🞏 Yes

🞏 No

If YES, which AAN risk factors were identified? (check all that apply)

🞏 Caregiver report

🞏 History of crashes and citations

🞏 Driving < 60 miles a week

🞏 Situational avoidance

🞏 Aggression, Impulsivity

🞏 Conditions endorsed

Which of these medical conditions were endorsed during the eligibility screening? (check all that apply)

🞏 Alcohol or other substance use

🞏 Obstructive sleep apnea

🞏 Narcolepsy

🞏 Field cuts

🞏 Low visual acuity even after correction

🞏 Macular degeneration

🞏 Diabetic retinopathy

🞏 Spinal cord injury

🞏 Multiple sclerosis

🞏 Brain injury

🞏 Syncope

🞏 Parkinson's

🞏 Seizure

🞏 Stroke

Date PI is notified and sent relevant info about participant (incl. identified AAN risk factors) _______________

Date PI sent email to participant _______________

Explain the outcome of the PI contacting the participant _______________

Explain the outcome of the PI contacting the participant _______________

If participant shared evaluation results, upload it here _______________
