# Supplementary material for: The Advancing Understanding of Transportation Options (AUTO) study: design and methods of a multi-center study of decision aid for older drivers
Source: Inj Epidemiol. 2021 May 3;8:23. doi: 10.1186/s40621-021-00310-4 (PMC8088834; doi:10.1186/s40621-021-00310-4)

**Appendix 4. Cognitive Concern Form**

***INSTRUCTIONS:*** To be completed by the RA when there is concern for the participant (driver) having cognitive decline.

**Survey**

In your own words, please describe the primary reason(s) for completing a cognitive concern document for this participant. Please include any relevant information.

___________________________________________________________________________

___________________________________________________________________________

___________________________________________________________________________

| **GENERAL** | | | | |  |  | |
| --- | --- | --- | --- | --- | --- | --- | --- |
| Were the participant’s answers inconsistent with the questions more than 50 % of the time? | | | | | Yes | No | |
| Did the questions have to be repeated 3 or more times because of lack of understanding (excluding loss of hearing)? | | | | | Yes | No | |
| Were questions left unanswered because it was impossible to obtain an answer (excluding indecisiveness)? | | | | | Yes | No | |
| Did it take at least twice as long as a usual interview? | | | | | Yes | No | |
| **DRIVING** | | | | |  |  | |
| Did the participant report if it was suggested by someone, like a doctor or family member, that they limit or stop driving? | | | | | Yes | No | |
| If YES, what was the outcome of the discussion? ______________________________  If YES, what triggered the discussion? ______________________________ | | | | |  |  | |
| Did the participant report any accidents in the last year? | | | | | Yes | No | |
| Did the participant report being pulled over by the police regardless of whether or not they received a ticket? | | | | | Yes | No | |
| Did the participant report receiving any tickets in the last year (e.g. red light camera, photo radar)? | | | | | Yes | No | |
| Has the participant decreased their driving? | | | | |  |  | |
|  | | | | |  |  | |
| **COGNITIVE** | | | | |  |  | |
| **Time-point** | **5-Min MoCA Score** | **Administered?** | | | **If yes, date** | | |
| Screening |  | -- | | |  | | |
| T2 |  | Yes No | | |  | | |
| T3 |  | Yes No | | |  | | |
| T4 |  | Yes No | | |  | | |
| T5 |  | Yes No | | |  | | |
| **Additional cognitive tests** | | |  | **Date administered** | | | |
| Was RBANS 80 or below? | | | Yes No |  | | | |
| Was Trails B age-corrected Z-score -1.5 or below? | | | Yes No |  | | | |
| Was BTACT Adjusted Composite Average of Z scores -1.5 or below? | | | Yes No |  | | | |
| Was Oral Trail Making Test Trial B Z Score -1.5 or below | | | Yes No |  | | | |
| **BEHAVIORAL** | | |  |  | | | |
| Did the participant have to be refocused frequently to the survey questions or tasks? | | | | | | Yes | No |
| If yes, how often? | | | | | |  |  |
| Did the participant tell you something repeatedly as if it were the first time it was told to you? | | | | | | Yes | No |
| If yes, how often? | | | | | |  |  |
| Did the participant tell you something repeatedly as if it were the first time it was told to you? | | | | | | Yes | No |
| If yes, how often? | | | | | |  |  |
| Did the participant have multiple word-finding difficulties when speaking? | | | | | | Yes | No |
| If yes, how often?____________________ | | | | | |  |  |
| **MEDICAL RECORD REVIEW** | | | | | |  |  |
| Does the participant have a diagnosis of dementia? | | | | | | Yes | No |
| **STUDY CONSENT** | | | | | |  |  |
| Did the participant give consent for permission to contact their PCP about their driving safety? | | | | | | Yes | No |

**Summary**

Date of PI review: ____/____/_____

PI summary: ______________________________________________________

PI action taken:

🞏 Contact and discussion with participant

🞏 Contact and discussion with PCP

🞏 Contact and discussion with someone other than their PCP

🞏 Participant withdrawn from study

🞏 No action


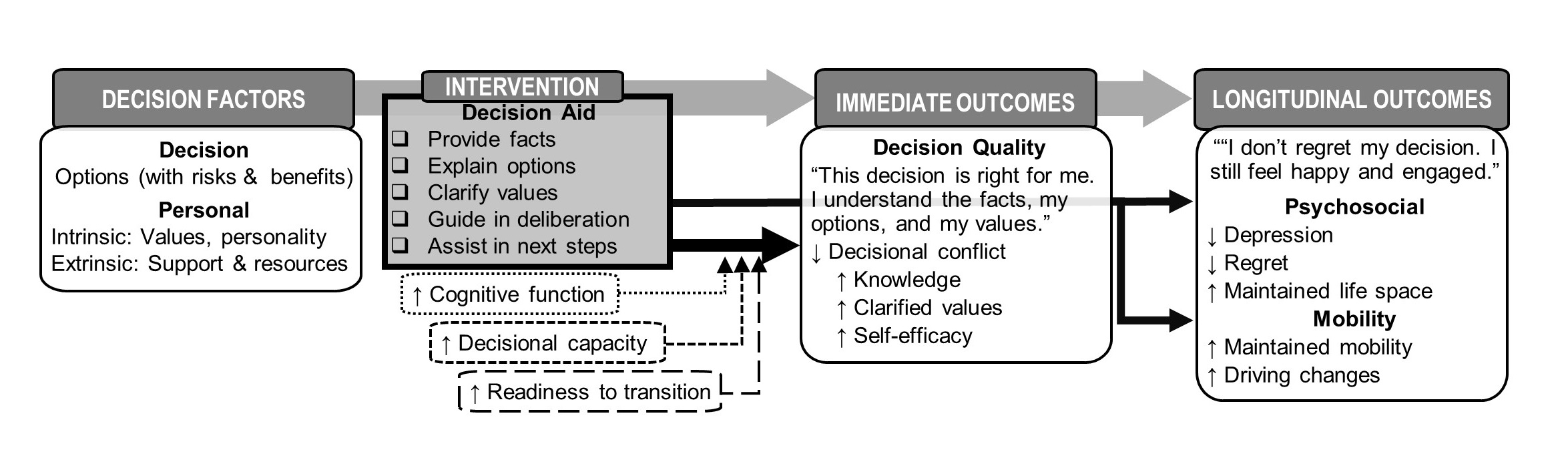

Supplement: Supplementary file 2 — Additional file 2: Cognitive Concern Form. [file 40621_2021_310_MOESM2_ESM.docx]
